# Metastasis to the parotid region as an initial presentation of renal cell carcinoma: A case report

**DOI:** 10.3892/ol.2013.1110

**Published:** 2013-01-07

**Authors:** CHAI YANLAN, SONG LIPING, CHE SHAOMIN, LIU ZI

**Affiliations:** Cancer Center, First Hospital of Xi’an Jiaotong University, Shaan’xi, Xi’an 710061, P.R. China

**Keywords:** renal cell carcinoma, metastasis, parotid region, therapy development

## Abstract

Distant metastasis of renal cell carcinoma (RCC) to the parotid region is extremely rare, particularly as an initial presentation. In the present study, we report a rare case of parotid region metastasis from RCC as an initial presentation in a 44-year-old female who presented with a painless lump in the right parotid region. Investigation revealed RCC in the left renal region and metastasis to the right iliac area. A radical nephrectomy was performed but the patient refused any further treatment. After seven months, the patient reappeared with systemic multiple metastases, with the exception of previous metastases that were enlarging significantly. On admission, interleukin-2 and local radiotherapy were administered. However, oral mucositis occurred. Targeted therapy with sunitinib was recommended.

## Introduction

Metastases of malignancies to the parotid region are relatively infrequent (21–42% of all malignant tumors) and originate primarily from head and neck squamous cell carcinoma and melanoma of the skin (1). Metastases of an infraclavicular origin are rare (0.16–4%) (1,2).

Renal cell carcinoma (RCC) is known for its high propensity for early metastasis and ≤1/3 of patients diagnosed with RCC present with metastatic disease at the time of diagnosis (3). Although RCC may metastasize to any organ system, its metastasis to the maxillofacial area is a relatively rare phenomenon. This study describes a patient whose first clinical presentation of RCC was parotid region metastasis. To the best of our knowledge, this case is the first documented example of such large metastasis of RCC.

The study was approved by the Ethics Committee of The First Affiliated Hospital of Medical College of Xi’an Jiaotong University, Shaan’xi, Xi’an, China, and written informed consent was obtained from the patient.

## Case report

**Figure 1:**
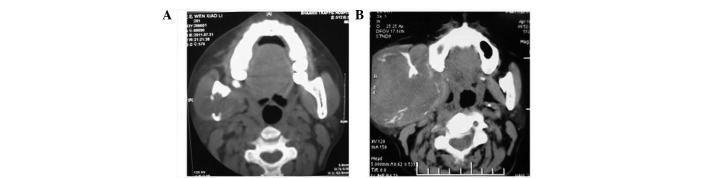
Computed tomography (CT) scan of the head and neck revealing a mass in the right parotid region. (A) Seven months previously: The mass is ∼4×2.5 cm and is accompanied by a slightly osteolytic destruction of the right mandibular branch. (B) At present: The enlarged mass is ∼12.5 cm and is accompanied by serious osteolytic destruction of the right mandibular branch.

**Figure 2:**
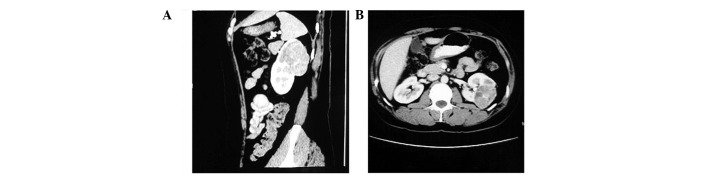
(A) Sagittal and (B) coronal computed tomography (CT) scan demonstrating the mass of the left kidney.

**Figure 3:**
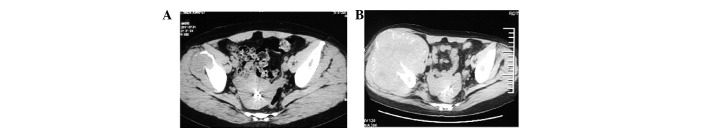
Computed tomography (CT) scan of the pelvic cavity revealing a mass in the right iliac area. (A) Seven months previously: The mass is ∼3 cm and is accompanied with a slightly osteolytic destruction of the right iliac bone. (B) At present: The enlarged mass is ∼13.5 cm and is accompanied with a serious osteolytic destruction of the right iliac bone.

**Figure 4:**
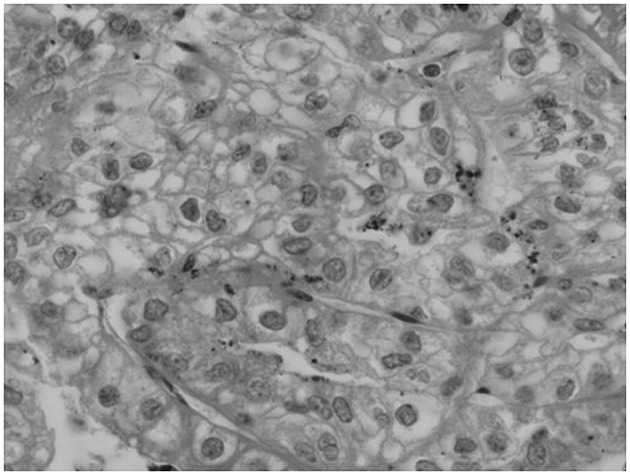
Histological analysis revealed a typical morphology of a middle-grade (grade 2) clear-cell renal cell carcinoma, which confirmed the patient’s tumor to be a primary neoplasm. Hematoxylin and esosin (H&E); magnification, ×1,000.

A 44-year-old female visited our hospital due to the presence of a painless mass in her parotid region. The patient denied all genitourinary symptoms and was otherwise well. Laboratory studies revealed normal hemogram, urinalysis and blood ureanitrogen results. The results of a complete ear, nose and throat examination were also normal. The head and neck computed tomography (CT) scan revealed a 4×2.5-cm mass in the right parotid region accompanied by an osteolytic destruction of the right mandibular branch (Fig. 1A). A roentgenogram of the chest was observed to be normal. Further examination by an abdominal CT scan revealed a 6.5-cm mass in the left kidney, suggesting RCC (Fig. 2), and a mass in the right iliac area accompanied by a slightly osteolytic destruction of the right iliac bone (Fig. 3A). A radical nephrectomy was performed. The histological analysis revealed a renal clear cell carcinoma (Fig. 4) and confirmed the renal tumor to be the primary neoplasm. The patient was then discharged from the hospital, as she did not accept any further treatment.

**Figure 5:**
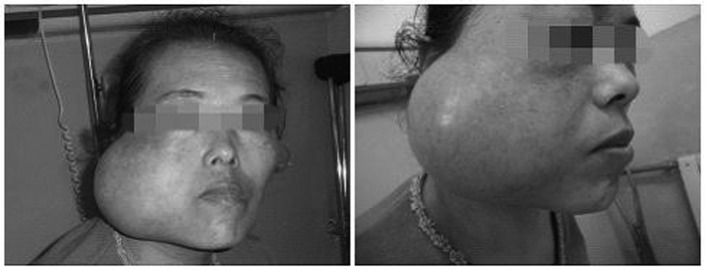
Ball-like mass in the right parotid region.

After seven months, the patient presented with the mass in the right parotid region that had enlarged to a ball-like size and was causing a difficulty in opening the mouth. On physical examination, positive findings consisted of a firm mass measuring ∼12×13 cm in the parotid region (Fig. 5). The mass demonstrated no tenderness but had local heat. The CT scan revealed that the masses in the right parotid region and the right iliac area had enlarged to 12.5 and 13.5 cm, respectively, and were accompanied by serious osteolytic destruction of the right mandibular branch and the right iliac bone, respectively (Figs. 1B and 3B). Furthermore, liver metastasis, multiple bone metastases and bilateral multiple metastatic lesions in the lungs were identified. The patient was administered interleukin (IL)-2 and bisphosphonates, and received local radiotherapy to the right parotid region and pelvic cavity. However, after three weeks, oral mucositis occurred. Due to unbearable pain, the patient did not accept further radiotherapy. Therefore, targeted therapy with sunitinib was recommended; however, the patient refused this treatment due to its high cost. The patient was treated with Chinese medicine and supportive care. Further follow-up continues.

## Discussion

In adults, RCC constitutes 2–3% of all malignancies, accounting for ∼90% of all primary renal tumors, with an incidence peak in the sixth decade of life (4,5). RCC are hypervascular tumors with a high expression of vascular endothelial growth factor (VEGF), the VEGF receptor (VEGFR), the platelet-derived growth factor (PDGF) receptor and basic fibroblast growth factor (bFGF) (6). Cancer cells are also known to have a good adaptive potential in a diverse array of microenvironments, giving rise to the high metastatic potential of RCC (7). Additionally, as the kidney receives ∼25% of the circulating blood volume per minute, the main mechanism of systemic metastases of RCC is hematogenous metastasis. The most common sites for RCC metastasis are the lungs (45.2%) and bone (29.5%), followed by the lymph nodes (21.8%) and the liver (20.3%) (8). Metastasis to the head and neck region are less common, comprising 14–16% of all cases (9). The current patient who initially presented with metastasis to the parotid region, which then developed into systemic multiple metastases, was a rare case.

Throughout the past decade, improvements in the understanding of the molecular pathways implicated in the pathogenesis of RCC has led to a marked expansion in the treatment options available to patients with metastatic RCC (mRCC). Previously, systemic treatment was limited to cytokine therapy with IL-2 or interferon (IFN), as mRCC is largely resistant to chemotherapy (10). However, in patients with mRCC, cytokine therapy is correlated with low response and high toxicity rates (10). In recent years, identification of VEGF, VEGFR and the mammalian target of rapamycin (mTOR) as dysregulated signaling pathways in the development and progression of RCC has led to the rapid development of novel molecular targeted therapies. Thus far, six targeted therapies, including sorafenib, sunitinib, bevacizumab (in combination with IFN-γ), temsirolimus, everolimus and pazopanib, have been evaluated in randomized, controlled phase III clinical trials of patients with mRCC and approved by the US Food and Drug Administration (FDA) for the management of mRCC (11). Each of these new agents has demonstrated a significant clinical benefit and fewer detrimental side-effects, leading to a better quality of life for patients (11–17). New targeted agents with novel mechanisms of action are also being studied, including histone deacetylase inhibitors, angiopoietin/tyrosine protein kinase receptor (TIE-2) inhibitors and carbonic anhydrase IX inhibitors (11).

Recent developments have raised the question of whether patients benefit most from combinatorial or sequential therapy of targeted agents. At present, sequential therapy with targeted agents is the standard of care (18). Combinatorial therapy strategies have not yet been demonstrated to be beneficial, with a number of combinations exhibiting excessive toxicity with marginal or inferior efficacy compared with that observed with the sequential use of agents (19). Ongoing investigations concerning the biomarkers that predict severe adverse events and the response of an individual patient to different targeted therapies will lead to a more personalized approach to treating mRCC (20,21). Additionally, novel immunological therapies are currently being developed to treat mRCC, including those that block cytotoxic T-lymphocyte antigen 4 (CTLA4) (11). Despite the recent progress, mRCC remains a disease with no curative therapy and further research is required.
